# 5-{[(3*R*,5a*S*,6*R*,8a*S*,9*R*,10*S*,12*R*,12a*R*)-3,6,9-Trimethyl­perhydro-3,12-ep­oxy-1,2-dioxepino[4,3-*i*]isochromen-10-yl]oxymeth­yl}benzene-1,3-diol

**DOI:** 10.1107/S1600536809002050

**Published:** 2009-01-23

**Authors:** Waseem Gul, Paulo Carvalho, Ahmed Galal, Mitchell A. Avery, Mahmoud A. El Sohly

**Affiliations:** a El Sohly Laboratories, Inc, 5 Industrial Park Drive, Oxford, MS 38655, USA; b Department of Medicinal Chemistry, University of Mississippi, 417 Faser Hall, University, MS 38677, USA; c National Center for Natural Products Research, Department of Pharmaceutics, School of Pharmacy, University of Mississippi, University, MS 38677, USA; d Department of Chemistry and Biochemistry, University of Mississippi, University, MS 38677, USA

## Abstract

The title compound, C_22_H_30_O_7_, is a fused five-ring system that is of inter­est for its anti­cancer and anti­malarial activity. The six-membered C_6_ and C_5_O rings display chair conformations. The six-membered C_3_O_3_ ring containing the ether and per­oxy functionalities has a distorted boat conformation, with a C—O—O—C torsion angle of 42.6 (1)° for the per­oxy group. The seven-membered C_6_O ring has a distorted boat-type conformation, while the seven-membered C_5_O_2_ ring has a very distorted chair-type conformation. The structure contains inter­molecular O—H⋯O and O—H⋯(O,O) bonds that link the mol­ecules into sheets parallel to the (100) planes.

## Related literature

For the crystallographic analysis of artemisinin, see: Lisgarten *et al.* (1998 ▶). For anti­malarial and anti­tumor activity of artemisinin, see Beekman *et al.* (1997 ▶, 1998 ▶); Pu *et al.* (1995 ▶); Zheng (1994 ▶).
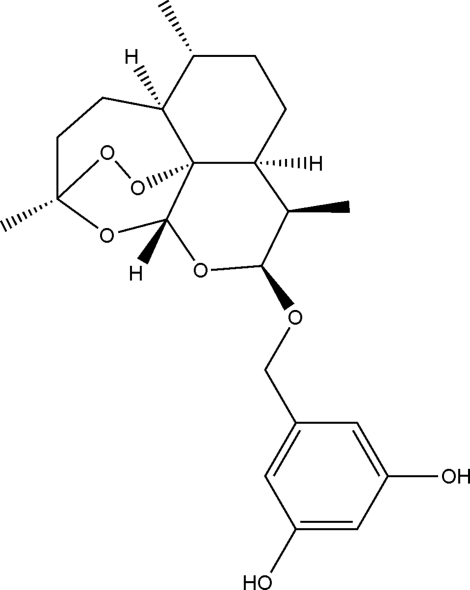


## Experimental

### 

#### Crystal data

C_22_H_30_O_7_
*M* *_r_* = 406.46
Monoclinic, 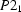

*a* = 10.3088 (2) Å
*b* = 10.2844 (2) Å
*c* = 10.3218 (3) Å
β = 113.14 (1)°
*V* = 1006.29 (9) Å^3^
*Z* = 2
Cu *K*α radiation
μ = 0.82 mm^−1^
*T* = 100 (2) K
0.23 × 0.15 × 0.08 mm

#### Data collection

Bruker APEXII CCD diffractometer
Absorption correction: none
15943 measured reflections
3623 independent reflections
3604 reflections with *I* > 2σ(*I*)
*R*_int_ = 0.019

#### Refinement

*R*[*F* ^2^ > 2σ(*F* ^2^)] = 0.026
*wR*(*F* ^2^) = 0.069
*S* = 1.08
3623 reflections
267 parameters
1 restraint
H-atom parameters constrained
Δρ_max_ = 0.25 e Å^−3^
Δρ_min_ = −0.22 e Å^−3^
Absolute structure: Flack (1983 ▶), 1623 Friedel pairs
Flack parameter: 0.04 (10)

### 

Data collection: *APEX2* (Bruker, 2005 ▶); cell refinement: *SAINT* (Bruker, 2005 ▶); data reduction: *SAINT*; program(s) used to solve structure: *SHELXS97* (Sheldrick, 2008 ▶); program(s) used to refine structure: *SHELXL97* (Sheldrick, 2008 ▶); molecular graphics: *SHELXTL* (Sheldrick, 2008 ▶); software used to prepare material for publication: *ORTEP-3 for Windows* (Farrugia, 1997 ▶).

## Supplementary Material

Crystal structure: contains datablocks I, global. DOI: 10.1107/S1600536809002050/bi2336sup1.cif

Structure factors: contains datablocks I. DOI: 10.1107/S1600536809002050/bi2336Isup2.hkl

Additional supplementary materials:  crystallographic information; 3D view; checkCIF report

## Figures and Tables

**Table 1:** Hydrogen-bond geometry (Å, °)

*D*—H⋯*A*	*D*—H	H⋯*A*	*D*⋯*A*	*D*—H⋯*A*
O6—H6⋯O7^i^	0.82	1.99	2.7279 (14)	150
O7—H7⋯O2^ii^	0.82	2.18	2.8409 (13)	138
O7—H7⋯O4^ii^	0.82	2.22	2.9269 (13)	144

Symmetry codes: (i) 
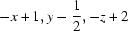
; (ii) 
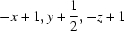
.

**Fig. 1.**
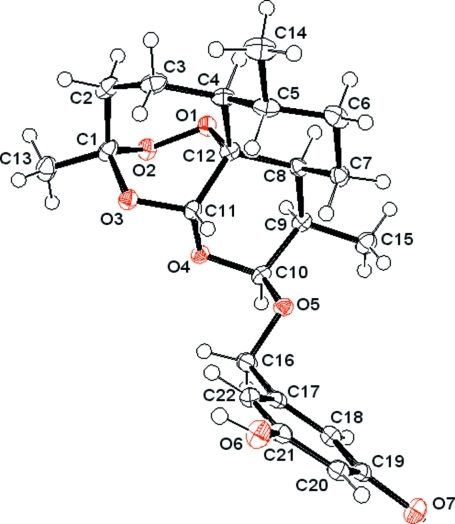
Molecular structure of the title compound with displacement ellipsoids drawn at the 50% probability level for non-H atoms.

## supplementary crystallographic information

### Comment

The title compound, a new dihydroartemisinin acetal monomeric derivative, has been prepared by reacting dihydroartemisinin with 1,3-dihydroxy-5-hydroxymethyl benzene using BF_3_.Et_2_O as the coupling catalyst. The compound exhibits anti-malarial, anti-cancer, and antiinfective activity.

### Experimental

To a stirred solution of dihydroartemisinin (120 mg, 0.42 mmol) in dry ether (40 ml) was added dry 3,5-dihydroxybenzyl alcohol (28 mg), followed by BF_3_.OEt_2_ (32 ml). Stirring was continued for two and half hours, after which time the reaction was quenched by addition of 10 ml of 2% aqueous solution of NaHCO_3_. The reaction mixture was then diluted with ether (50 ml), and transferred to a separatory funnel and extracted with ether (3 × 30 ml). The ether fractions were pooled, washed with water, and dried over Na_2_SO_4_. Removal of ether under reduced pressure left an oily crude product, which was purified over Si gel column using a gradient of EtOAc in hexanes (15% to 26%) to yield a colorless solid (35 mg, 28%).

### Refinement

All H atoms were visible in difference maps, but were placed geometrically and treated as riding atoms for refinement, with the following constraints: C—H = 0.93 Å, *U*_iso_(H) = 1.2*U*_eq_(C) for C*sp*^2^, C—H = 0.98 Å, *U*_iso_(H) = 1.2*U*_eq_(C) for CH, C—H = 0.97 Å, *U*_iso_(H) = 1.2*U*_eq_(C) for CH_2_, C—H = 0.96 Å, *U*_iso_(H) = 1.5*U*_eq_(C) CH_3_, O—H = 0.82 Å, *U*_iso_(H) = 1.5*U*_eq_(O) for OH.

### Crystal data

**Table:**

C_22_H_30_O_7_	*F*(000) = 436
*M**_r_* = 406.46	*D*_x_ = 1.341 Mg m^−^^3^
Monoclinic, *P*2_1_	Cu *K*α radiation, λ = 1.54178 Å
Hall symbol: P 2yb	Cell parameters from 9920 reflections
*a* = 10.3088 (2) Å	θ = 4.3–68.9°
*b* = 10.2844 (2) Å	µ = 0.82 mm^−^^1^
*c* = 10.3218 (3) Å	*T* = 100 K
β = 113.14 (1)°	Plate, colourless
*V* = 1006.29 (9) Å^3^	0.23 × 0.15 × 0.08 mm
*Z* = 2	

### Data collection

**Table:**

Bruker APEXII CCD diffractometer	3604 reflections with *I* > 2σ(*I*)
Radiation source: fine-focus sealed tube, Siemens KFF Cu 2 K90	*R*_int_ = 0.019
graphite	θ_max_ = 69.3°, θ_min_ = 4.7°
φ and ω scans	*h* = −12→12
15943 measured reflections	*k* = −11→12
3623 independent reflections	*l* = −12→12

### Refinement

**Table:**

Refinement on *F*^2^	Secondary atom site location: difference Fourier map
Least-squares matrix: full	Hydrogen site location: inferred from neighbouring sites
*R*[*F*^2^ > 2σ(*F*^2^)] = 0.026	H-atom parameters constrained
*wR*(*F*^2^) = 0.069	*w* = 1/[σ^2^(*F*_o_^2^) + (0.0416*P*)^2^ + 0.199*P*] where *P* = (*F*_o_^2^ + 2*F*_c_^2^)/3
*S* = 1.08	(Δ/σ)_max_ < 0.001
3623 reflections	Δρ_max_ = 0.25 e Å^−^^3^
267 parameters	Δρ_min_ = −0.22 e Å^−^^3^
1 restraint	Absolute structure: Flack (1983), 1623 Friedel pairs
Primary atom site location: structure-invariant direct methods	Flack parameter: 0.04 (10)

### Special details

**Table:**

Geometry. All e.s.d.'s (except the e.s.d. in the dihedral angle between two l.s. planes) are estimated using the full covariance matrix. The cell e.s.d.'s are taken into account individually in the estimation of e.s.d.'s in distances, angles and torsion angles; correlations between e.s.d.'s in cell parameters are only used when they are defined by crystal symmetry. An approximate (isotropic) treatment of cell e.s.d.'s is used for estimating e.s.d.'s involving l.s. planes.
Refinement. Refinement of *F*^2^ against ALL reflections. The weighted *R*-factor *wR* and goodness of fit *S* are based on *F*^2^, conventional *R*-factors *R* are based on *F*, with *F* set to zero for negative *F*^2^. The threshold expression of *F*^2^ > σ(*F*^2^) is used only for calculating *R*-factors(gt) *etc*. and is not relevant to the choice of reflections for refinement. *R*-factors based on *F*^2^ are statistically about twice as large as those based on *F*, and *R*-factors based on ALL data will be even larger.

### Fractional atomic coordinates and isotropic or equivalent isotropic displacement parameters (Å^2^)

**Table:**

	*x*	*y*	*z*	*U*_iso_*/*U*_eq_	
O4	0.24620 (9)	0.53498 (9)	0.32197 (9)	0.01671 (19)	
O1	−0.02356 (10)	0.56559 (9)	0.09293 (9)	0.0177 (2)	
O6	0.41598 (11)	0.67491 (10)	1.04175 (10)	0.0238 (2)	
H6	0.3735	0.6057	1.0194	0.036*	
O5	0.34731 (9)	0.70855 (9)	0.47383 (9)	0.01720 (19)	
O3	0.07779 (9)	0.40591 (9)	0.33999 (9)	0.0184 (2)	
O7	0.71639 (10)	0.95263 (9)	0.93260 (10)	0.0213 (2)	
H7	0.7608	0.9578	0.8820	0.032*	
O2	0.02421 (10)	0.42912 (9)	0.10142 (9)	0.0191 (2)	
C18	0.58553 (13)	0.79300 (13)	0.75672 (13)	0.0177 (3)	
H18	0.6249	0.8215	0.6946	0.021*	
C1	−0.01197 (14)	0.36064 (14)	0.20186 (14)	0.0204 (3)	
C20	0.56095 (14)	0.81251 (13)	0.97930 (13)	0.0178 (3)	
H20	0.5828	0.8554	1.0646	0.021*	
C10	0.31061 (13)	0.66001 (13)	0.33627 (13)	0.0172 (3)	
H10	0.3973	0.6505	0.3195	0.021*	
C8	0.06357 (14)	0.75832 (13)	0.22778 (13)	0.0166 (3)	
H8	0.0018	0.7965	0.1376	0.020*	
C4	−0.13890 (13)	0.62313 (15)	0.24865 (13)	0.0193 (3)	
H4	−0.2079	0.6579	0.1599	0.023*	
C11	0.11686 (13)	0.53590 (13)	0.34304 (13)	0.0158 (3)	
H11	0.1353	0.5713	0.4368	0.019*	
C12	0.00544 (13)	0.62013 (13)	0.23280 (13)	0.0161 (3)	
C22	0.43232 (13)	0.64640 (13)	0.81424 (14)	0.0184 (3)	
H22	0.3703	0.5763	0.7905	0.022*	
C7	0.05456 (14)	0.84730 (14)	0.34327 (14)	0.0198 (3)	
H7A	0.1191	0.8161	0.4346	0.024*	
H7B	0.0834	0.9345	0.3305	0.024*	
C5	−0.13896 (13)	0.71641 (15)	0.36552 (13)	0.0221 (3)	
H5	−0.0685	0.6848	0.4551	0.026*	
C15	0.28159 (15)	0.88607 (14)	0.23539 (15)	0.0233 (3)	
H15A	0.3043	0.9221	0.3276	0.035*	
H15B	0.2171	0.9428	0.1660	0.035*	
H15C	0.3662	0.8771	0.2183	0.035*	
C14	−0.28261 (14)	0.71858 (18)	0.37886 (15)	0.0297 (3)	
H14A	−0.2827	0.7861	0.4430	0.044*	
H14B	−0.2986	0.6362	0.4137	0.044*	
H14C	−0.3561	0.7348	0.2881	0.044*	
C17	0.48996 (13)	0.69005 (13)	0.72052 (13)	0.0174 (3)	
C6	−0.09417 (15)	0.85158 (15)	0.34012 (14)	0.0231 (3)	
H6A	−0.0971	0.9106	0.4123	0.028*	
H6B	−0.1590	0.8836	0.2493	0.028*	
C16	0.44908 (14)	0.62693 (14)	0.57838 (14)	0.0200 (3)	
H16A	0.5318	0.6160	0.5566	0.024*	
H16B	0.4087	0.5418	0.5788	0.024*	
C9	0.21337 (13)	0.75281 (13)	0.22646 (13)	0.0178 (3)	
H9	0.2017	0.7167	0.1347	0.021*	
C21	0.46825 (13)	0.70848 (14)	0.94374 (13)	0.0186 (3)	
C19	0.62111 (13)	0.85242 (13)	0.88701 (14)	0.0173 (3)	
C3	−0.19196 (14)	0.48755 (15)	0.26635 (14)	0.0237 (3)	
H3A	−0.1469	0.4632	0.3649	0.028*	
H3B	−0.2925	0.4932	0.2432	0.028*	
C2	−0.16688 (14)	0.37942 (15)	0.17856 (14)	0.0234 (3)	
H2A	−0.2207	0.3982	0.0797	0.028*	
H2B	−0.2024	0.2986	0.2004	0.028*	
C13	0.02745 (16)	0.22097 (15)	0.19089 (15)	0.0262 (3)	
H13A	−0.0393	0.1835	0.1055	0.039*	
H13B	0.0267	0.1729	0.2703	0.039*	
H13C	0.1201	0.2176	0.1899	0.039*	

### Atomic displacement parameters (Å^2^)

**Table:**

	*U*^11^	*U*^22^	*U*^33^	*U*^12^	*U*^13^	*U*^23^
O4	0.0160 (4)	0.0163 (5)	0.0179 (4)	−0.0009 (3)	0.0068 (4)	−0.0024 (3)
O1	0.0225 (5)	0.0173 (5)	0.0130 (4)	−0.0007 (4)	0.0067 (4)	−0.0007 (4)
O6	0.0285 (5)	0.0254 (5)	0.0221 (5)	−0.0052 (4)	0.0149 (4)	0.0005 (4)
O5	0.0169 (4)	0.0187 (5)	0.0140 (4)	0.0006 (4)	0.0039 (3)	−0.0015 (3)
O3	0.0215 (5)	0.0181 (5)	0.0140 (4)	−0.0030 (4)	0.0051 (4)	−0.0005 (3)
O7	0.0232 (5)	0.0217 (5)	0.0232 (5)	−0.0058 (4)	0.0138 (4)	−0.0047 (4)
O2	0.0234 (5)	0.0183 (5)	0.0170 (4)	−0.0016 (4)	0.0094 (4)	−0.0024 (4)
C18	0.0191 (6)	0.0192 (7)	0.0165 (6)	0.0030 (5)	0.0088 (5)	0.0022 (5)
C1	0.0246 (7)	0.0215 (7)	0.0146 (6)	−0.0075 (5)	0.0072 (5)	−0.0028 (5)
C20	0.0185 (6)	0.0203 (7)	0.0145 (6)	0.0029 (5)	0.0063 (5)	−0.0002 (5)
C10	0.0165 (6)	0.0197 (7)	0.0162 (6)	−0.0034 (5)	0.0074 (5)	−0.0032 (5)
C8	0.0192 (6)	0.0167 (7)	0.0134 (6)	0.0021 (5)	0.0060 (5)	0.0014 (5)
C4	0.0149 (6)	0.0279 (7)	0.0146 (6)	0.0008 (5)	0.0052 (5)	0.0002 (5)
C11	0.0177 (6)	0.0158 (7)	0.0149 (6)	−0.0018 (5)	0.0075 (5)	−0.0022 (5)
C12	0.0176 (6)	0.0202 (7)	0.0109 (6)	−0.0015 (5)	0.0060 (5)	−0.0016 (5)
C22	0.0147 (6)	0.0170 (7)	0.0220 (6)	−0.0001 (5)	0.0055 (5)	0.0005 (5)
C7	0.0229 (7)	0.0170 (7)	0.0185 (6)	0.0017 (5)	0.0072 (5)	−0.0011 (5)
C5	0.0178 (6)	0.0329 (8)	0.0153 (6)	0.0039 (6)	0.0063 (5)	0.0005 (6)
C15	0.0253 (7)	0.0239 (8)	0.0213 (7)	−0.0053 (6)	0.0097 (6)	−0.0003 (6)
C14	0.0222 (7)	0.0464 (10)	0.0225 (7)	0.0053 (7)	0.0112 (6)	−0.0023 (7)
C17	0.0150 (5)	0.0182 (7)	0.0174 (6)	0.0050 (5)	0.0045 (5)	0.0015 (5)
C6	0.0249 (7)	0.0269 (8)	0.0177 (6)	0.0080 (6)	0.0086 (5)	−0.0015 (6)
C16	0.0190 (6)	0.0200 (7)	0.0190 (6)	0.0032 (5)	0.0053 (5)	0.0005 (5)
C9	0.0195 (6)	0.0209 (7)	0.0139 (6)	−0.0036 (5)	0.0075 (5)	−0.0016 (5)
C21	0.0159 (6)	0.0215 (7)	0.0189 (6)	0.0038 (5)	0.0075 (5)	0.0044 (5)
C19	0.0147 (6)	0.0164 (7)	0.0199 (6)	0.0019 (5)	0.0058 (5)	0.0023 (5)
C3	0.0178 (6)	0.0346 (8)	0.0196 (6)	−0.0045 (6)	0.0082 (5)	0.0006 (6)
C2	0.0227 (7)	0.0279 (8)	0.0190 (6)	−0.0101 (6)	0.0075 (5)	−0.0011 (5)
C13	0.0335 (7)	0.0225 (8)	0.0213 (7)	−0.0056 (6)	0.0094 (6)	−0.0031 (6)

### Geometric parameters (Å, °)

**Table:**

O4—C10	1.4280 (16)	C11—H11	0.980
O4—C11	1.4328 (15)	C22—C17	1.3936 (19)
O1—C12	1.4660 (15)	C22—C21	1.3937 (19)
O1—O2	1.4785 (13)	C22—H22	0.930
O6—C21	1.3644 (16)	C7—C6	1.5214 (19)
O6—H6	0.820	C7—H7A	0.970
O5—C10	1.4092 (15)	C7—H7B	0.970
O5—C16	1.4423 (16)	C5—C6	1.520 (2)
O3—C11	1.3930 (17)	C5—C14	1.5403 (17)
O3—C1	1.4384 (15)	C5—H5	0.980
O7—C19	1.3730 (16)	C15—C9	1.5266 (19)
O7—H7	0.820	C15—H15A	0.960
O2—C1	1.4191 (16)	C15—H15B	0.960
C18—C19	1.3888 (18)	C15—H15C	0.960
C18—C17	1.3936 (19)	C14—H14A	0.960
C18—H18	0.930	C14—H14B	0.960
C1—C13	1.509 (2)	C14—H14C	0.960
C1—C2	1.5308 (19)	C17—C16	1.5052 (18)
C20—C21	1.3845 (19)	C6—H6A	0.970
C20—C19	1.3874 (18)	C6—H6B	0.970
C20—H20	0.930	C16—H16A	0.970
C10—C9	1.5190 (18)	C16—H16B	0.970
C10—H10	0.980	C9—H9	0.980
C8—C7	1.5344 (18)	C3—C2	1.520 (2)
C8—C9	1.5506 (17)	C3—H3A	0.970
C8—C12	1.5512 (18)	C3—H3B	0.970
C8—H8	0.980	C2—H2A	0.970
C4—C3	1.535 (2)	C2—H2B	0.970
C4—C5	1.5415 (19)	C13—H13A	0.960
C4—C12	1.5598 (16)	C13—H13B	0.960
C4—H4	0.980	C13—H13C	0.960
C11—C12	1.5289 (17)		
			
C10—O4—C11	113.64 (9)	C6—C5—H5	107.7
C12—O1—O2	111.75 (9)	C14—C5—H5	107.7
C21—O6—H6	109.5	C4—C5—H5	107.7
C10—O5—C16	112.09 (10)	C9—C15—H15A	109.5
C11—O3—C1	113.63 (10)	C9—C15—H15B	109.5
C19—O7—H7	109.5	H15A—C15—H15B	109.5
C1—O2—O1	109.72 (9)	C9—C15—H15C	109.5
C19—C18—C17	118.94 (12)	H15A—C15—H15C	109.5
C19—C18—H18	120.5	H15B—C15—H15C	109.5
C17—C18—H18	120.5	C5—C14—H14A	109.5
O2—C1—O3	108.18 (10)	C5—C14—H14B	109.5
O2—C1—C13	105.17 (11)	H14A—C14—H14B	109.5
O3—C1—C13	107.17 (11)	C5—C14—H14C	109.5
O2—C1—C2	112.33 (11)	H14A—C14—H14C	109.5
O3—C1—C2	109.88 (10)	H14B—C14—H14C	109.5
C13—C1—C2	113.79 (11)	C22—C17—C18	120.53 (12)
C21—C20—C19	119.65 (12)	C22—C17—C16	120.35 (12)
C21—C20—H20	120.2	C18—C17—C16	119.12 (12)
C19—C20—H20	120.2	C5—C6—C7	109.99 (11)
O5—C10—O4	110.67 (10)	C5—C6—H6A	109.7
O5—C10—C9	111.50 (10)	C7—C6—H6A	109.7
O4—C10—C9	110.18 (10)	C5—C6—H6B	109.7
O5—C10—H10	108.1	C7—C6—H6B	109.7
O4—C10—H10	108.1	H6A—C6—H6B	108.2
C9—C10—H10	108.1	O5—C16—C17	108.73 (10)
C7—C8—C9	113.15 (11)	O5—C16—H16A	109.9
C7—C8—C12	112.23 (10)	C17—C16—H16A	109.9
C9—C8—C12	111.46 (10)	O5—C16—H16B	109.9
C7—C8—H8	106.5	C17—C16—H16B	109.9
C9—C8—H8	106.5	H16A—C16—H16B	108.3
C12—C8—H8	106.5	C10—C9—C15	111.45 (11)
C3—C4—C5	111.25 (11)	C10—C9—C8	112.57 (10)
C3—C4—C12	113.19 (11)	C15—C9—C8	113.79 (11)
C5—C4—C12	112.84 (10)	C10—C9—H9	106.1
C3—C4—H4	106.3	C15—C9—H9	106.1
C5—C4—H4	106.3	C8—C9—H9	106.1
C12—C4—H4	106.3	O6—C21—C20	115.57 (12)
O3—C11—O4	105.56 (10)	O6—C21—C22	124.17 (12)
O3—C11—C12	113.63 (10)	C20—C21—C22	120.26 (12)
O4—C11—C12	111.25 (10)	O7—C19—C20	116.06 (11)
O3—C11—H11	108.8	O7—C19—C18	122.91 (11)
O4—C11—H11	108.8	C20—C19—C18	121.03 (12)
C12—C11—H11	108.8	C2—C3—C4	116.22 (11)
O1—C12—C11	108.60 (10)	C2—C3—H3A	108.2
O1—C12—C8	104.45 (9)	C4—C3—H3A	108.2
C11—C12—C8	110.91 (10)	C2—C3—H3B	108.2
O1—C12—C4	106.09 (10)	C4—C3—H3B	108.2
C11—C12—C4	113.69 (10)	H3A—C3—H3B	107.4
C8—C12—C4	112.48 (11)	C3—C2—C1	114.21 (11)
C17—C22—C21	119.54 (12)	C3—C2—H2A	108.7
C17—C22—H22	120.2	C1—C2—H2A	108.7
C21—C22—H22	120.2	C3—C2—H2B	108.7
C6—C7—C8	111.64 (11)	C1—C2—H2B	108.7
C6—C7—H7A	109.3	H2A—C2—H2B	107.6
C8—C7—H7A	109.3	C1—C13—H13A	109.5
C6—C7—H7B	109.3	C1—C13—H13B	109.5
C8—C7—H7B	109.3	H13A—C13—H13B	109.5
H7A—C7—H7B	108.0	C1—C13—H13C	109.5
C6—C5—C14	111.72 (12)	H13A—C13—H13C	109.5
C6—C5—C4	109.92 (11)	H13B—C13—H13C	109.5
C14—C5—C4	112.02 (11)		

### Hydrogen-bond geometry (Å, °)

**Table:**

*D*—H···*A*	*D*—H	H···*A*	*D*···*A*	*D*—H···*A*
O6—H6···O7^i^	0.82	1.99	2.7279 (14)	150
O7—H7···O2^ii^	0.82	2.18	2.8409 (13)	138
O7—H7···O4^ii^	0.82	2.22	2.9269 (13)	144

Symmetry codes: (i) −*x*+1, *y*−1/2, −*z*+2; (ii) −*x*+1, *y*+1/2, −*z*+1.
